# Fatal Nosocomial MDR TB Identified through Routine Genetic Analysis and Whole-Genome Sequencing

**DOI:** 10.3201/eid2106.141903

**Published:** 2015-06

**Authors:** O. Martin Williams, Thomas Abeel, Nicola Casali, Keira Cohen, Alex S. Pym, Sarah B. Mungall, Christopher A. Desjardins, Anindo Banerjee, Francis Drobniewski, Ashlee M. Earl, Graham S. Cooke

**Affiliations:** Bristol Royal Infirmary, Bristol, UK (O.M. Williams); Delft University of Technology, Delft, the Netherlands (T. Abeel);; Broad Institute, Cambridge, Massachusetts, USA (T. Abeel, C.A. Desjardins, A.M. Earl);; Queen Mary University of London, London, UK (N. Casali);; Imperial College London, London (N. Casali, F. Drobniewski, G.S. Cooke);; K-Research Institute for TB/HIV, Durban, South Africa (K. Cohen, A.S. Pym);; University Hospitals Bristol NHS Foundation Trust, Bristol (S.B. Mungall);; University Hospital Southampton NHS Trust, Southampton, UK (A. Banerjee)

**Keywords:** MDR-TB, tuberculosis, nosocomial infection, antimicrobial resistance, health care workers, whole-genome sequencing, tuberculosis and other mycobacteria

**To the Editor:** In November 2012, a 44-year-old HIV-negative white man (patient 1) with fever, fatigue, and breathlessness sought care at a hospital in the United Kingdom. He had never traveled abroad but had biopsy-proven alcoholic cirrhosis. No acid-fast bacilli were seen on multiple samples, including ascitic fluid, and he received treatment for presumptive abdominal tuberculosis (TB). *Mycobacterium tuberculosis* was subsequently cultured after 12 days. His clinical condition deteriorated, and he died of multiorgan failure 44 days after admission. The cultured *M. tuberculosis* was subsequently confirmed as multidrug resistant (Technical Appendix Table).

Routine mycobacterial interspersed repetitive unit–variable-number tandem-repeat (MIRU-VNTR) testing was performed (*1*) (Technical Appendix Table). A matching MIRU-VNTR profile was identified from a 42-year-old South African–born, HIV-positive health care worker (patient 2) who had died in 2008 after admission to the same hospital. She has been described previously in detail because she had worked at Tugela Ferry hospital in KwaZulu-Natal, South Africa, which was associated with a 2005 outbreak of multidrug-resistant TB (MDR TB) and extensively drug-resistant TB (*2*,*3*) (Technical Appendix Figure 1). To ascertain whether these isolates could have matching MIRU-VNTR patterns by chance alone, we compared the MIRU-VNTR results with a national database of ≈11,745 isolates typed since the UK typing service began in 2010. Only 2 other isolates matched (from patients 3 and 4), originating from a UK hospital ≈100 miles away. Although both patients were HIV-positive health care workers from sub-Saharan Africa, no history of contact could be established with patients 1 or 2.

A review of admission records established that patients 1 and 2 were admitted to the same medical ward in 2008 for 8 days, suggesting a high probability of nosocomial transmission. The ward had a traditional “Nightingale” configuration with beds for male and female patients arranged dormitory-style. In 2009, patient 1 had been identified as a contact of patient 2 and was offered screening for latent infection but had failed to attend appointments and was not under regular medical follow-up. No other common contact was identified. The estimated time from known contact between patients 1 and 2 until the clinical presentation of patient 1 was 49 months.

Sequencing libraries from genomic DNA extracted from the 4 UK *M. tuberculosis* isolates that had matching MIRU-VNTR profiles were paired-end sequenced by using Illumina MiSeq (Illumina, San Diego, CA, USA). To investigate the origins of the infections, they were compared with 36 South Africa strains (including 1 from the Tugela Ferry outbreak [*4*]) sequenced by using Illumina HiSeq 2000 platforms.

For each sequenced strain, a random subset of reads was aligned at ≈100× coverage to the *M. tuberculosis* H37Rv reference genome by using BWA version 0.5.9-r16 (*5*). Pilon v1.5 (http://www.broadinstitute.org/software/pilon/) was run in variant discovery to generate a list of single-nucleotide polymorphisms (SNPs) and insertions and deletions. We estimated a phylogeny using RAxML v7.7.8 (*6*) using a general time reversible + gamma substitution model with 1,000 bootstrap replicates.

Pairwise comparison of whole-genome sequences from *M. tuberculosis* isolated from patients 1 and 2 found that the 2 sequences differed at only 4 SNPs (Table). Based on previous estimates of background mutations rates of 0.5 SNP/year (*7*), the pairwise distance between isolates from patient 1 and 2 increases confidence in the epidemiologic data implicating transmission >4 years earlier, although uncertainties exist around such estimates. Comparison between samples from patient pairs (1+2 vs. 3+4) found differences of 69–72 SNPs, which strongly argues against transmission between them.

**Table:** Pairwise distances between 2 pairs of *Mycobacterium tuberculosis* isolates from patients in the United Kingdom, an isolate from the 2005 Tugela Ferry outbreak in KwaZulu-Natal, South Africa (KZN605), and reference strain H37Rv

Isolate	Patient 1	Patient 2	KZN605	Patient 3	Patient 4	H37Rv
Patient 1	0					
Patient 2	4	0				
KZN605	21	24	0			
Patient 3	84	80	87	0		
Patient 4	87	83	90	2	0	
H37Rv	862	862	887	849	830	0

In comparison with isolates sampled from KwaZulu-Natal (Technical Appendix Figure 1), isolates from patients 1 and 2 were closely related to a strain associated with the Tugela Ferry outbreak (KZN605; Technical Appendix Figure 2). Isolates from patients 3 and 4 were less closely related to isolates from the Tugela Ferry outbreak but were closely related to other isolates circulating within the region, consistent with the hypothesis that both infections originally occurred within South Africa.

This investigation illustrates the power of current technology to inform our understanding of the links in MDR TB transmission between low- and high-incidence areas. Whole-genome sequencing of pathogens is becoming part of routine practice for establishing transmission and resistance patterns (*8*). The greater certainty it brings to transmission data can provide evidence to justify more active policies of screening and isolation as part of infection control. The nosocomial transmission described here is consistent with the fact that a person with pulmonary TB (patient 2) was managed on an open ward before being put into respiratory isolation and had not been previously screened by occupational health services.

Recent data reviewing MDR TB transmission in the United Kingdom before 2007 did not identify cases of nosocomial transmission during that period (*9*). However, the emergence of MDR TB in regions of high HIV prevalence is relatively recent (*10*), and the cases described here suggest that increased vigilance for TB and MDR TB among migrating health care workers might be required.

Technical Appendix Characteristics of 4 clinical isolates of multidrug-resistant *Mycobacterium tuberculosis*; isolates sequenced from KwaZulu-Natal, South Africa; and phylogenetic representation of isolates collected from the United Kingdom and KwaZulu-Natal.
