# Supplementary material for: Fatal Nosocomial MDR TB Identified through Routine Genetic Analysis and Whole-Genome Sequencing
Source: Emerg Infect Dis. 2015 Jun;21(6):1082–4. doi: 10.3201/eid2106.141903 (PMC4451893; doi:10.3201/eid2106.141903)
Supplement: Technical Appendix — Characteristics of 4 clinical isolates of multidrug-resistant Mycobacterium tuberculosis; isolates sequenced from KwaZulu-Natal, South Africa; and phylogenetic representation of isolates collected from the United Kingdom and KwaZulu-Natal. [file 14-1903-Techapp-s1.pdf]

Article DOI: http://dx.doi.org/10.3201/eid2106.141903

Fatal Nosocomial MDR-TB Identified through Routine Genetic Analysis and Whole-Genome Sequencing

Technical Appendix

Technical Appendix Table. Characteristics of 4 clinical isolates from patients with multidrug-resistant tuberculosis*

Patient	MIRU-VNTR	RIF	rpoB	rpoB	INH	katG	mabA†	PZA	pncA	EMB	embB	STR	gidB‡	gidB‡
1	224331432615324 33241328	R	L452P§	H1028R	R	S315T	–8(t>a)	R	ins¶	R	M306V	S	130 bp del	R
2	224331432615324	R	L452P§	H1028R	R	S315T	–8(t>a)	R	ins¶	R	M306V	–	130 bp del	R
3	22433143261532-3324132-	S	–	–	R	S315T	–	S	–	S	–	R	–	L26S
4	22433143261532-33241328	S	–	–	R	S315T	–	S	–	S	–	R	–	L26S

*EMB, ethambutol; INH, isoniazid; MIRU-VNTR, mycobacterial interspersed repetitive units–variable-number tandem-repeat; PZA, pyrazinamide; R, resistant; RIF, rifampin; S, sensitive; STR, streptomycin.
†Promoter mutation, cross-resistance to thioamides.
§*Escherichia coli* L533P
‡No resistance single-nucleotide polymorphisms in rpsL or rrs.
¶1 bp insertion in codon 153.

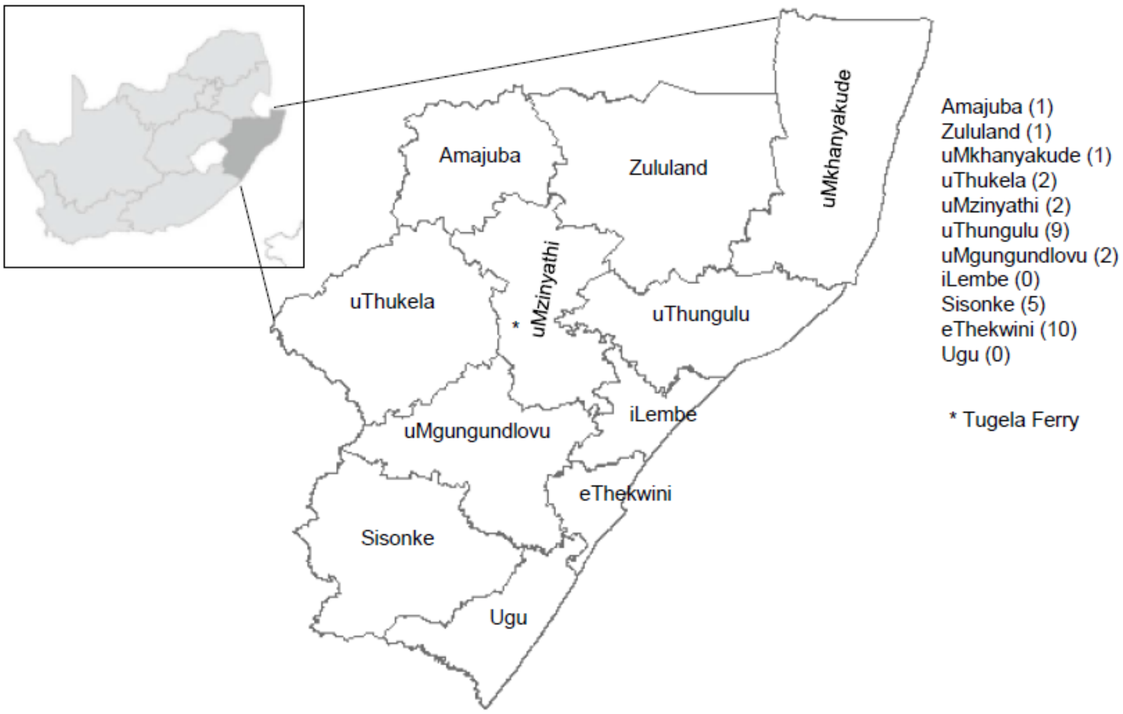

Technical Appendix Figure 1. Isolates sequenced from KwaZulu-Natal (KZN), South Africa, with number from each district sampled as part of KZNSUR project. Inset: Map of South Africa with KZN shaded

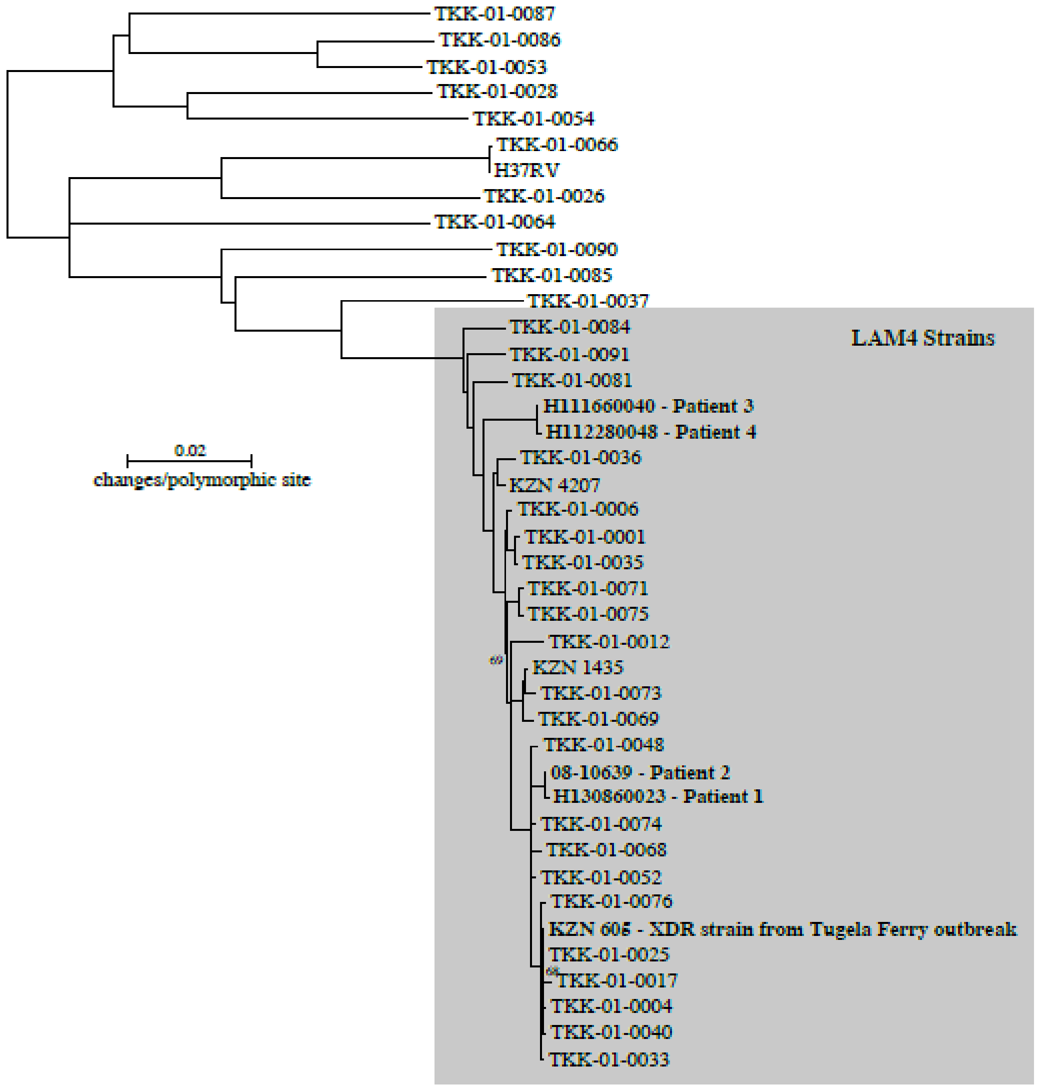

Technical Appendix Figure 2. Phylogenetic representation of isolates collected from the United Kingdom (patients 1–4) and KZN (33) Nodes with bootstrap values <80 are indicated. Sequencing data were submitted to the Sequence Read Archive with identifiers: PRJNA198182, PRJNA198181, PRJNA198148, PRJNA198149, PRJNA198124, PRJNA198185, PRJNA198180, PRJNA198186, PRJNA198176, PRJNA198108, PRJNA198103, PRJNA198130, PRJNA183521, PRJNA198168, PRJNA227150, PRJNA198163, PRJNA198172, PRJNA198106, PRJNA183515, PRJNA198122, PRJNA227148, PRJNA198135, PRJNA198128, PRJNA198147, PRJNA198169, PRJNA198143, PRJNA198113, PRJNA198167, PRJNA198170, PRJNA198131, PRJNA227149, PRJNA198179, PRJNA198132, PRJNA183519, PRJNA198161, PRJNA198159 ERS568284, ERS568285, ERS568286, ERS 568287
